# Giant retroperitoneal well differentiated liposarcoma: A case report and literature review

**DOI:** 10.1016/j.ijscr.2023.108679

**Published:** 2023-08-22

**Authors:** Tilahun Habte Nureta, Wongel Tena Shale, Tewodros Deneke Belete

**Affiliations:** a Jimma University College of Public Health and Medical Sciences, Department of General Surgery, Jimma, Ethiopia; b Jimma University College of Public Health and Medical Sciences, GI oncology surgery Unit, Jimma, Ethiopia; c Jimma University College of Public Health and Medical Sciences, Department of Pathology, Jimma, Ethiopia

**Keywords:** Retroperitoneal tumors (RPTs), Giant retroperitoneal liposarcoma, Well differentiated retroperitoneal liposarcoma (WDLS), Case report

## Abstract

**Introduction and importance:**

The most prevalent type of primary retroperitoneal tumors is soft tissue sarcoma (STS). Liposarcoma accounts for 40 % of retroperitoneal tumors (Mack, 1995). Retroperitoneal liposarcoma accounts for 12 % to 40 % of all liposarcomas (Vijay and Ram, 2015). They typically present with advanced disease and often carry a poor prognosis. Because of their rarity and anatomic location, these malignant tumors can cause a diagnostic dilemma and present several therapeutic challenges (Vijay and Ram, 2015).

**Case presentation:**

A 48-year-old male patient presented to our gastrointestinal oncology clinic with a 2-year history of abdominal discomfort, weight loss and steadily growing abdominal swelling. A soft, rubbery lobulated mass with a positive “slippage sign” was palpable over all the quadrants of the abdomen. CT scan conclusion was retroperitoneal lipoma with internal enhancing nodular components. During laparotomy, a fatty mass measuring 55*60*22 cm and weighing 14 kg was excised. Histopathologic report showed a well differentiated liposarcoma (WDLS).

**Clinical discussion:**

Giant retroperitoneal liposarcoma (RPL) is exceedingly rare. Liposarcomas have diverse MRI and CT appearances due to the various subtypes. WDLS are difficult to identify from lipomas before surgery. Histopathology is the only way to provide a reliable diagnosis; therefore en block resection is the recommended approach when malignancy cannot be ruled out.

**Conclusion:**

Although imaging with ultrasound, CT, and MRI can aid in the diagnosis, differentiating WDLS from lipoma pre-operatively can be difficult, and en block resection is the recommended approach when malignancy cannot be ruled out.

## Highlights

• Giant retroperitoneal liposarcoma (RPL) (>30 cm in diameter/ >20 kg) is exceedingly rare with only a couple of reports in the literature.
• In this article we describe a case of a 48- year- old male patient who had a massive retroperitoneal well differentiated liposarcoma measuring 55*60*22 cm.
• Well differentiated retroperitoneal liposarcomas are difficult to identify from lipomas before surgery.
• Histopathology is the only way to provide a reliable diagnosis; therefore en block resection is recommended when malignancy cannot be ruled out.
• Following surgery, regular follow-up is advised, as recurrence is a possibility.

## 1 Introduction

RPTs are tumors that develop in the retroperitoneal region typically from mesenchymal tissues, including adipose tissue, muscles, the lymphatic system, blood vessels, cartilage, and bones [1,2]. Most primary RPTs, between 70 and 80 % of them, are cancerous. Soft tissue sarcoma (STS) is the most common primary RPT type, while liposarcomas are the most common among STS [3]. Nearly 40 % of retroperitoneal tumors are liposarcomas [3].

A malignant tumor that arises from adipose tissue is called a liposarcoma. Anywhere that has fat tissue is where it can happen [4]. According to the WHO, based on histology, there are four different forms of liposarcomas. The types are well differentiated, dedifferentiated, pleomorphic, and myxoid, the former of which is more frequently seen in the retroperitoneal cavity and the latter of which frequently originates in the extremities [5]. Patients often present with advanced disease and frequently have a bad prognosis since the retroperitoneal area is vast and they typically remain asymptomatic until the mass becomes large enough to exert pressure on surrounding structures or infiltrate crucial organs. These malignant tumors can present a number of therapeutic difficulties due to their rarity and anatomical position. They can also pose diagnostic difficulties [4].

Well-differentiated retroperitoneal liposarcomas are slow-growing, low-grade tumors that frequently reach large size before exhibiting symptoms and being found, raising the risk of surgery and the possibility of dedifferentiation [6]. It can be difficult to identify lipomas from WDLPS prior to surgery because both have a lot of fat and little soft tissue and look identical, despite the fact that imaging modalities, in particular CT-scan and MRI, are crucial to make a diagnosis in retroperitoneal tumors [7]. As a result, en-block resection based on oncologic principles is the therapy option if malignancy cannot be ruled out. We discuss the case of a 48-year-old male patient who had been experiencing vague abdominal pain for two years that was first diagnosed as primary retroperitoneal lipoma. The final histology report revealed that the patient had well-differentiated liposarcoma. The work has been reported in line with the SCARE criteria [41].

## 2 Case presentation

A 48-year-old male patient presented to our gastrointestinal oncology clinic with a 2-year history of abdominal discomfort and steadily growing abdominal swelling. He had experienced significant weight loss since the onset of the illness. He complained of occasional early satiety but no nausea or vomiting. He was constipated seldom but he experienced no significant changes in his bowel habits or rectal bleeding. He has no known chronic medical or surgical disease. He does not consume alcohol or smoke cigarettes. He does not use any illicit or prescribed drugs.

### 2.1 Personal and family history

No noteworthy personal or family history was reported by the patient.

### 2.2 Physical examination

**Fig. 1:**
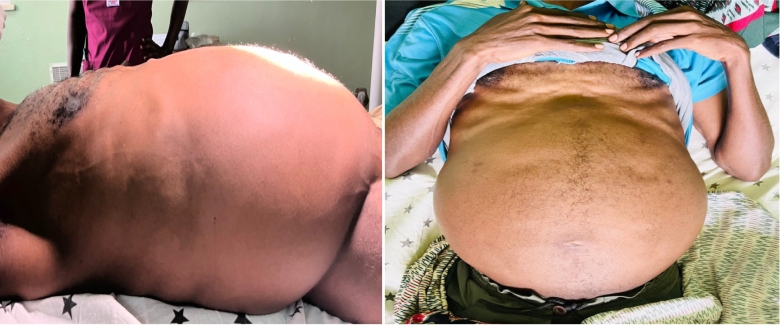
The lateral (left) and antero-posterior (right) view of patient's distended abdomen on inspection.

The patient appeared to be chronically ill (emaciated). His vital signs were all within normal limits. His conjunctivae were pink. The abdomen was severely distended, with bulging flanks (Fig. 1). A soft, rubbery lobulated mass with a positive “slippage sign” was palpable over all the quadrants of the abdomen. A firm nodularity of 8*10 cm was felt within the mass in the right lower quadrant, and it was mobile. It was difficult to illicit signs of free fluid collection.

### 2.3 Laboratory investigations

He was investigated with complete blood count (CBC), WBC = 4.4 × 10^3^/μL, HGB = 16.5 g/dl, PLT = 176 × 10^3^/μL.

Serum albumin level: 4.2 g/dl and serum electrolytes and organ function tests, which were all within normal limits.

### 2.4 Imaging

#### 2.4.1 Abdominopelvic ultrasound

Huge echoreflective intraabdominal mass with homogenous surface filling the whole abdomen; there is focal hypoechoic lesion within the mass measuring 5 cm*7.2 cm. No free fluid seen.

Index: Mesenteric lipoma.

#### 2.4.2 Abdominopelvic CT-scan

Huge fat attenuated intraabdominal mass measuring 30 cm*36 cm*39 cm, internal enhancing two solid components seen within the mass having different tissue attenuation, the larger one measures 7.8 cm*8 cm. Bowel loops are pushed posterior to the mass.

**Fig. 2:**
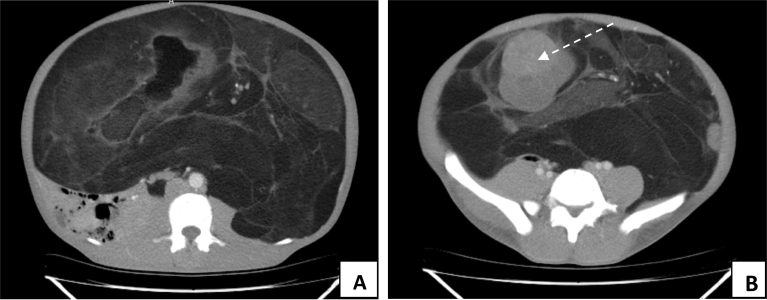
A: Computed tomography imaging showing a giant homogenous mass, mainly consisting of fatty tissue measuring 30 cm*36 cm*39 cm with thin septa, pushing the peritoneal contents such as bowel loops posteriorly and with internal enhancing nodular components), B: Thin arrow depicting large enhancing component).

**Fig. 3:**
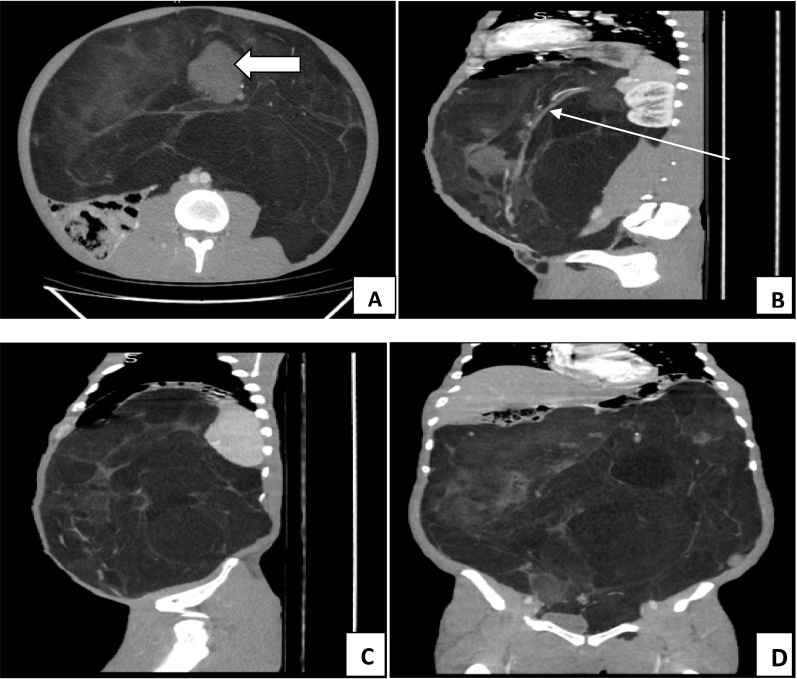
A: Thick arrow depicting the smaller solid component, B: Thin arrow showing the displaced left Ureter running along the right postero-lateral aspect of the mass, C: Sagittal view of the mass, D: Coronal view of the mass.

Index: Intra-abdominal fat attenuated mass likely retroperitoneal lipoma with internal enhancing nodular components (Fig. 2), (Fig. 3).

## 3 Final diagnosis

We diagnosed the tumor as a huge retroperitoneal lipoma based on its clinical symptoms and imaging evidence indicating an adipose origin. However, due to its large size and enhancing solid components, the possibility of malignancy could not be ignored. In addition, an MRI is typically needed to adequately define soft tissue masses, but one was not available to us.

## 4 Treatment

After completing pre- operative assessment and optimization, three units of whole blood were prepared. Informed consent was obtained from the patient and he was taken to the OR. After administering prophylactic antibiotics, Ceftriaxone 1 g stat 30 min prior to skin incision, Patient was put under general anesthesia, positioned supine and exploratory laparatomy was performed.

**Fig. 4:**
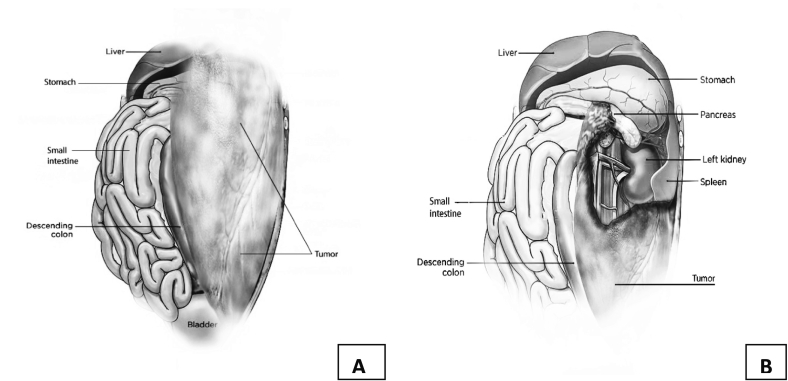
Surgical schema depicting the spatial orientation and origin of tumor in the abdominal cavity, A; Solid illustration, B; Sectioned illustration.

**Fig. 5:**
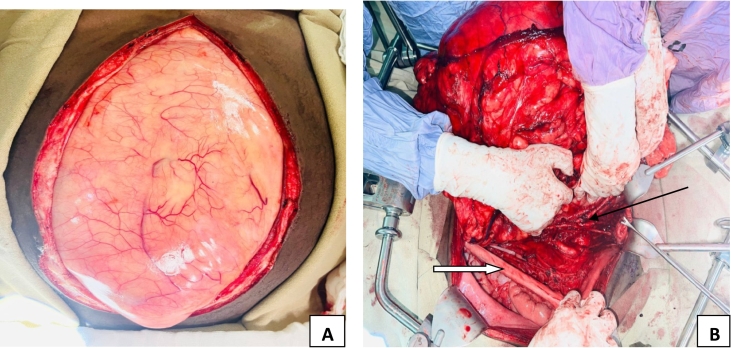
A: Lipomatous mass filling the entire abdominopelvic cavity, B: Small bowel and left colon pushed to the right (thick white arrow), Infracolic retroperitoneal vessels separated from the mass (thin black arrow).

Intra-operative finding: During laparotomy, a bulky yellow to orange mass arising from the left retroperitoneum within a translucent capsule and almost filling the entire abdominopelvic cavities was discovered. Sigmoid, descending, and transverse colon were pushed to the right side of the abdomen, the left ureter and gonadal vessels were adherent on the right side of the mass, but it did not involve the Gerota's fascia, so the kidney was preserved. The bulk went through the mesocolon and was mildly adhered to the mesocolic vessels. (Fig. 4) The small bowel and its mesentery were completely pushed into the right upper quadrant and had no connection to the mass. Since the tumor was inframesocolic, the stomach, duodenum, and the superior mesenteric vessels were not in contact with it. We carefully dissected away the transverse colon, and the body and tail of the pancreas from the mass because they were in close proximity to it. The mass was resting on major inframesocolic retroperitoneal vessels namely common ileac, external and internal ileac vessels as well as the aortic bifurcation. The dome of the bladder was adherent to the tumor. (Fig. 5).

**Fig. 6:**
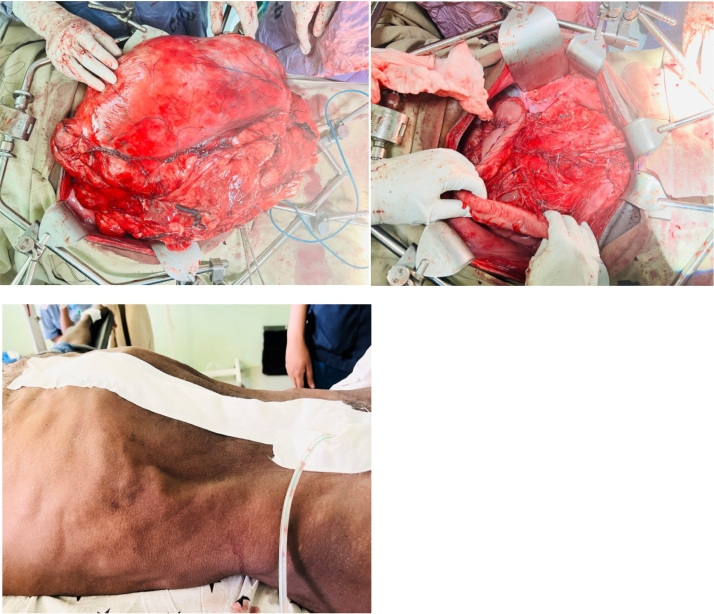
Completely excised retroperitoneal lipoma, abdominal closure with drain insitu.

Done: We did not do en bloc resection with the bowel because the tumor was grossly benign. The mesocolic vasculature was carefully isolated from the mass. We dissected away the left ureter, the gonadal vessels and the retroperitoneal vessels (left Common, internal and external ileac vessels) and The mass was completely excised, measuring 55*60*22 cm and weighing 14 kg, with all other structures preserved except for some part of the dome of the bladder which was excised with the tumor. The bladder was repaired in two layers. Hemostasis was secured, and the abdomen closed after a drain was left in place (Fig. 5), (Fig. 6).

Post-op course: He had an uneventful post operative recovery. Because he began ambulating soon after surgery, he was not given chemical DVT prophylaxis. On post-op day 2, enteral feeding was initiated and tolerated. The abdominal drain was removed after 6 days, when the output had greatly decreased, and the patient was discharged on the eighth postoperative day.

### 4.1 Histopathology report

**Fig. 7:**
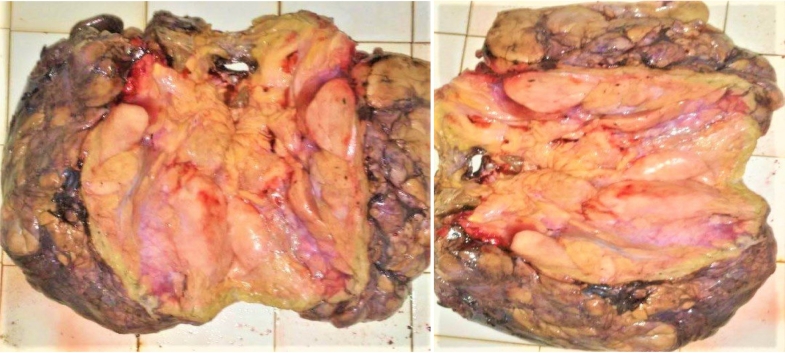
Gross pathologic appearance of a huge well differentiated primary retroperitoneal liposarcoma.

**Fig. 8:**
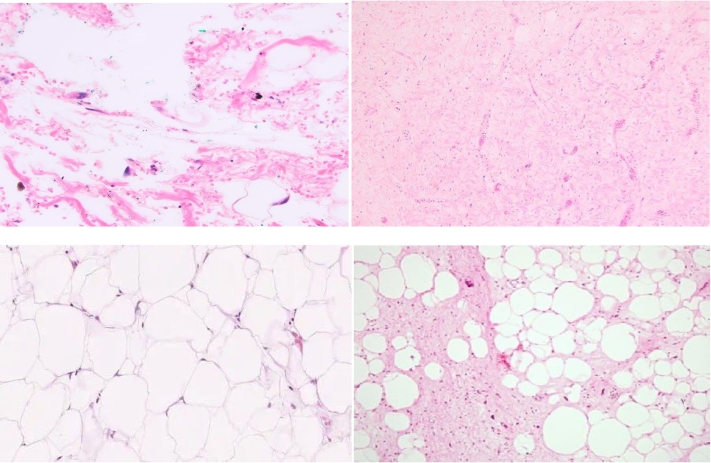
Microscopic appearance of a well differentiated primary retroperitoneal liposarcoma mature fat with low cellularity and variable sized adipocytes, bands of fibrotic stroma containing spindle cells having enlarged, hyperchromatic nuclei. There are focuses of lipoblasts with markedly atypical, few mitotic features.

Section showed mature fat with low cellularity and variable sized adipocytes, bands of fibrotic stroma containing spindle cells having enlarged, hyperchromatic nuclei. There are focuses of lipoblasts with markedly atypical, few mitotic features. (Fig. 7), (Fig. 8) All surgical margins were adequately free. The negative margins measured ≥10 mms on all sides.

***He was observed at the outpatient clinic for a year after the operation. His imaging tests and clinical examinations were both clean. He'll keep up the constant monitoring**.*

## 5 Discussion

Liposarcoma makes up 45 % of retroperitoneal soft-tissue sarcomas in adults, making it the most common kind [8]. Although the underlying risk factors are not fully understood, multiple studies have suggested a probable connection to certain chromosal abnormalities. Liposarcomas can occur anywhere in the body [6]. The American Cancer Society lists a number of factors that can increase your risk of developing liposarcoma, including prior radiation, specific familial cancer syndromes, trauma to the lymphatic system, and exposure to hazardous chemicals. It is crucial to realize that lipomas, which are completely benign, do not give rise to liposarcomas [9]. There isn't much of an age or sex predilection [10].

According to the World Health Organization's (WHO) 2002 classification, liposarcoma is a complex tumor, and categorizing it into three subtypes helps to best describe its pathogenesis. These include pleomorphic liposarcomas (PLS), myxoid and round cell liposarcomas (MLS and RCL), and well- and dedifferentiated liposarcomas (WDLPS/DDLPS). A diagnostic large marker and a ring chromosome which impacts unchecked cell proliferation will be used to identify WDLPS/DDLPS. In MCL and RCL, reciprocal translocation between chromosomes 12 and 16 occurs, leading to the activation of a number of downstream pathways, including PPARgamma2 and C/EBPalpha, which promotes cell proliferation [11]. Pleomorphic liposarcoma is the least understood and most difficult of them. Mutations in the p53, NF1, and RB1 tumor suppressor pathways result in numerous gains and this explains the tumor's aggressiveness [12].

The average diameter and weight of RPL during diagnosis is 20–25 cm and 15–20 kg. Although there is no consensus regarding the precise definition of giant RPL, it is defined by several literatures as an RPL of >30 cm in diameter or with weight of >20 kg. Giant retroperitoneal liposarcomas are exceedingly rare [27]. Only 17 cases with the diameter of 30 cm and above have been reported (Table 1).

**Table 1:** Summary of reported cases of retroperitoneal liposarcoma measuring above 30 cm diameter in adults.

Reference	Age	Sex	Tumor size	Histopathologic type	Extent of resection
Yol 1998 [12]	63	M	35 in diameter	Myxoid	R0
McCallum 2006 [13]	47	F	50 × 48 × 45	Poorly differentiated	R1
Clar 2009 [14]	66	M	47 × 25 × 42	Well differentiated	R0
Hashimoto 2010 [15]	41	M	45 × 40 × 30	Poorly differentiated	R0
Bansal 2012 [16]	52	M	40 × 35 × 35	Mixed	R0
De Nardi 2012 [17]	40	M	50 × 49 × 35	Well differentiated	R0
Sharma 2013 [18]	60	F	47 × 40 × 25	Well differentiated	R0
Zhang 2015 [19]	48	F	30 × 20 × 15	Myxoid	R0
Caizzone 2015 [20]	64	F	42 × 37 × 18	Pleomorphic liposarcoma with myxoid areas	R0
Hazen 2017 [21]	64	M	40 in diameter	Poorly differentiated	R0
Oh 2016 [22]	71	F	45 × 30 × 11	Well differentiated	R0
Zeng 2017 [23]	45	M	65 × 45 × 30	Well differentiated	R0
Herzberg 2019 [24]	75	M	35 × 29 × 20.5	Poorly differentiated	R0
LOCURTO 2019 [26]	69	M	30 × 21 × 33	well differentiated	R0
Xu, Chi 2020 [25]	65	M	37 × 32 × 26.5	Well differentiated	R0
Kanthala 2021 [28]	40	F	50 × 40 × 40 cm	De-differentiated liposarcoma (DDL) with undifferentiated pleomorphic sarcoma (UPS) features	- 12 months, no recurrence
Montenegro 2019 [29]	65	F	30 × 23 × 16 cm	pleomorphic liposarcoma	R0 6 month follow-up,no recurrence

Retroperitoneal tumors are typically asymptomatic in the early stages because of the enormous amount of space in the retroperitoneum. Once the tumors have reached massive sizes, local tissue and organ compression may take place, leading to obstructive urinary/bowel symptoms such lower extremities edema, early satiety, and abdominal discomfort. Like in the case of our patient, who had been asymptomatic throughout the illness except from a minor, nebulous abdominal ache. The clinical symptoms are often ambiguous [30]. Imaging tests are therefore essential in the diagnosis of malignant tumors.

The differential diagnosis of a retroperitoneal lesion might be narrowed down by the presence of fat on imaging. When investigating abdominal masses, ultrasound is frequently used as the first imaging method. An additional diagnostic method for retroperitoneal tumors is the use of CT and MRI. On computed tomography, fat typically attenuates minimally (10 to 100 HU). On CT and MRI, they show up as uniform fat-containing masses with thin septa. Because it has a higher sensitivity for displaying microscopic fat using chemical shift imaging and macroscopic fat using fat-suppression techniques, magnetic resonance imaging is a better imaging modality [31].

Retroperitoneal liposarcomas are difficult to identify from lipomas before surgery. Both lipomas and WDLPS have a large amount of fat and minimal soft tissue and have similar appearances on CT and MRI, making it difficult to differentiate lipomas from well-differentiated liposarcomas preoperatively [7] On CT, classic lipomas generally have Hounsfield unit measurements between −65 and − 120 (apart from the thin and wispy soft- tissue density septa, although on occasion septa may be thick and nodular). WDLS resemble lipomas on MR and CT images. Although a lesion with fully homogeneous fat signal intensity or density and only a few tiny, wispy septa can be presumed to be benign, they are difficult to conclusively distinguish from benign lipomas. These tumors are low-grade lesions that often contain >75 % of their volume in fat. To make the diagnosis, a thorough pathologic study of the tumor may be necessary. Compared to lipomas, fibrous septa may be thicker and more nodular. These tumors often recur if only marginally excised but do not metastasize [42].

Therefore, the final diagnosis rests on histopathological evaluation to assess mitotic activity, cellular atypia, necrosis and invasion [32]. Despite the fact that benign lipogenic tumors such as spindle cell/pleomorphic lipomas also exhibit lipoblasts, multivariate analysis in a study revealed that lipoblasts were statistically significant for the diagnosis of WDLS. Nuclear atypia was another finding that, according to univariate analysis, made a substantial difference in the differential diagnosis of WDLS. This trait is utilized to rule out the diagnosis of lipoma because nuclear atypia is not present in lipomas. In this study, nuclear atypia had a 100 % sensitivity for the diagnosis of WDLS. Additionally, FISH testing for MDM2 and CDK4 gene amplification has produced the most accurate results for WDLS diagnosis and is regarded as the gold standard for differentiating between WDLS and lipoma [43]. If malignancy cannot be ruled out, surgical exploration with thorough oncological resection is the treatment of choice [33]. There is currently no compelling evidence in the literature to support the need for a histological biopsy of a suspected retroperitoneal liposarcoma prior to therapy [34]. For patients with distant metastases or unresectable neoplasm, aspiration cytology is advised to inform preoperative radiation or chemotherapy planning [25]. Even though the WDLS is far more common than lipoma, the indolent course of disease progression, lack of symptoms, and benign imaging findings in this instance led us to assume the diagnosis was a primary retroperitoneal lipoma.

The main treatment for non-metastatic retroperitoneal liposarcoma is currently surgery. If local invasion is confirmed, the ultimate surgical strategy requires the removal of adjacent organs like the kidney and intestines as well as the complete surgical excision of the tumor with negative margins [25]. In 1988, Lewis et al. demonstrated the importance of a R0 resection [35]. The long-term 5- and 10-year survival rates without complete resection are, respectively, 16.7 % and 8.0 %, according to Zeng et al.'s review [23]. This supports the curative role of surgical excision in retroperitoneal sarcomas [24]. If the tumor is associated with non-resectable vital organs or has a higher risk of morbidity and mortality with total excision, preoperative radiation and/or chemotherapy may be explored to downstage the tumor [36]. Since no trials have shown a definite improvement in survival outcomes, the function of chemotherapy in the management of retroperitoneal liposarcoma is currently still up for debate [37].

This case has taught us that, even when a benign lipoma is clinically suspected, intraoperative tumor characteristics and subsequent judgment calls regarding the scope of resection are crucial. A wide excision should be performed if infiltrative growth is suspected because resection with negative margins (R0) is essential to the patient's prognosis in cases of liposarcoma [38,39]. Only tumors with a clearly defined margin should be excised simply. Preoperative determination of resectability based on CT scans is challenging, especially if the tumor mass is large. Tumor debulking for symptom relief is an option if oncological resection is not feasible. Excision should always be carried out by a skilled oncological surgeon in a high-volume center with experience in managing soft tissue sarcoma cases [38,39], as is the case at our facility. In our situation, the tumor appeared to be delineated and was soft and movable. There was no indication that adjacent tissue had been invaded.

One of the most crucial prognostic indicators is histopathology, which shows the characteristics of the tumor and the degree of differentiation. The five-year survival rate for well-differentiated liposarcoma in the retroperitoneum is around 90 %. Dedifferentiated, myxoid, and pleomorphic variant rates, on the other hand, range from 30 % to 75 %, with pleomorphic variant having the worst prognosis [36]. All liposarcoma subtypes are well-differentiated in about 45 % of cases. It is a locally aggressive tumor that has a slim chance of spreading to distant organs [36,40]. WDLS has a better prognosis than other histologic categories, but because the local aggressiveness makes recurrence more likely, careful and routine monitoring is advised. Our patient was kept under close surveillance after leaving the hospital. He has been observed at the outpatient clinic for a year, and so far there has not been a recurrence of the disease. His clinical examination, ultrasound, and CT scan results were unremarkable, and he had no complaints. The routine follow-up will carry on.

## 6 Conclusion

Giant retroperitoneal liposarcomas are very uncommon. Because of the peculiar nature of the retroperitoneum, it can grow to attain a massive size with no clinical signs, as demonstrated in the above instance. Although imaging with ultrasound, CT, and MRI can aid in the diagnosis, differentiating WDLS from lipoma pre-operatively can be difficult, and en block resection is the recommended approach when malignancy cannot be ruled out. Histopathology is the only way to provide a reliable diagnosis. Following surgery, regular follow-up is advised, as recurrence is a possibility.

## 7 Learning points

The important take away from this case report is that clinically and on CT/MRI, it is difficult to distinguish a benign retroperitoneal lipoma from a WDLS. As a result, even for benign disease, wide local excision using oncologic principles remains the mainstay of treatment.

## Registration of research studies

Not applicable.

## Consent

Written informed consent was obtained from the patient for publication of this case report and accompanying images. A copy of the written consent is available for review by the Editor-in-Chief of this journal on request.

## Guarantor

Tilahun Habte Nureta and Wongel Tena Shale are the guarantors of this study.

## Ethical approval

Case reports are exempted from ethical approval in Jimma University. As long as identifying patient information is removed and written consent is taken from the patient.

## Funding

There are no sources of funding to declare for this study.

## CRediT authorship contribution statement

Tilahun Habte Nureta contributed to writing of the manuscript, was the lead surgeon in performing the surgery, data collection and final follow up of the patient. First and Final draft of the article was written by Wongel Tena Shale, who also participated in the surgical treatment of the patient. Tewodros Belete Deneke did the histopathology examination and report. Critical revisions and final approval were made by all authors.

## Declaration of competing interest

None to declare.
